# Engineering muscle tissue for the fetus: getting ready for a strong life

**DOI:** 10.3389/fphar.2015.00053

**Published:** 2015-04-10

**Authors:** George J. Christ, Mevan L. Siriwardane, Paolo de Coppi

**Affiliations:** ^1^Wake Forest Institute for Regenerative MedicineWinston-Salem, NC, USA; ^2^Laboratory of Regenerative Therapeutics, Deptartment of Biomedical Engineering and Orthopaedic Surgery, University of VirginiaCharlottesville, VA, USA; ^3^Developmental Biology and Cancer Programme, UCL Institute of Child Health, Great Ormond Street HospitalLondon, UK

**Keywords:** skeletal muscle, tissue engineering, stem cells, biomaterials, regenerative medicine, congenital abnormalities, functional regeneration, animal models

## Abstract

Congenital malformations frequently involve either skeletal, smooth or cardiac tissues. When large parts of those tissues are damaged, the repair of the malformations is challenged by the fact that so much autologous tissue is missing. Current treatments require the use of prostheses or other therapies and are associated with a significant morbidity and mortality. Nonetheless, affected children have generally good survival rates and mostly normal schooling. As such, new therapeutic modalities need to represent significant improvements with clear safety profiles. Regenerative medicine and tissue engineering technologies have the potential to dramatically improve the treatment of any disease or disorder involving a lack of viable tissue. With respect to congenital soft tissue anomalies, the development of, for example, implantable muscle constructs would provide not only the usual desired elasticity and contractile proprieties, but should also be able to grow with the fetus and/or in the postnatal life. Such an approach would eliminate the need for multiple surgeries. However, the more widespread clinical applications of regenerative medicine and tissue engineering technologies require identification of the optimal indications, as well as further elucidation of the precise mechanisms and best methods (cells, scaffolds/biomaterials) for achieving large functional tissue regeneration in those clinical indications. In short, despite some amazing scientific progress, significant safety and efficacy hurdles remain. However, the rapid preclinical advances in the field bode well for future applications. As such, translational researchers and clinicians alike need be informed and prepared to utilize these new techniques for the benefit of their patients, as soon as they are available. To this end, we review herein, the clinical need(s), potential applications, and the relevant preclinical studies that are currently guiding the field toward novel therapeutics.

## Introduction

The majority of children affected by congenital malformations have a defect involving either skeletal, smooth or cardiac tissues. When large parts of those tissues are damaged, the repair of the malformations can be often challenged by the fact that autologous tissue is missing (de Coppi, 2013). Major cardiac anomalies, bladder exstrophy, omphaloceles, diaphragmatic hernia or long gap oesophageal atresia are only some of the situations in which we have to use prostheses or adopt solutions associated with a significant degree of morbidity and mortality. Since these children have, overall, good survival rates and mostly normal schooling, it is important to avoid using solutions that are not optimal, as this may limit their options in the future. Ideally, besides the usual elasticity and contractility proprieties, the implanted muscle, in contrast to implantation in post-adolescent individuals, should be able to grow with the fetus and/or into the postnatal life. To avoid a planned re-do surgery, absorbable materials should therefore be considered.

One classical example is congenital diaphragmatic hernia. Fetuses diagnosed with this malformation mostly receive a synthetic prosthesis at birth which allows the repair of the diaphragmatic defect. However, this patch will neither grow with the child, nor integrate with the native tissue, so there's more chance of muscle contraction with possible scoliosis, hernia recurrence or patch infections resulting in a poor quality of life for the child and his or her family (de Coppi and Deprest, 2012). For all these reasons many surgeons dealing with congenital malformations have been interested in tissue regeneration (Grikscheit et al., 2003; Atala et al., 2006; Kunisaki et al., 2006).

The possibility of making new tissue *in vitro* would indeed completely change the way we treat these children and transform their lives. The recent progress in tissue engineering (TE) and regenerative medicine (RM) has been possible due to improved understanding and utilization of the stem cells and biomaterials that are cornerstones for this field of medical science. Due their overt importance to the more widespread clinical applications of TE and RM technologies, both will be considered herein. We will begin with consideration of stem cells.

## Stem cells for TE and RM applications

Stem cells have developmental potentials varying from the totipotency of cells derived from the first few divisions of the fertilized egg to the unipotency of somatic cells present in peripheral tissue (Thomson et al., 1998; Pittenger et al., 1999). To regenerate large amounts of tissues, pluripotent cells would be ideal because they can be expanded and are able to generate any tissue (Thomson et al., 1998). However, they are still limited in their clinical use because, besides ethical concerns and immunogenicity, which have been partially overcome with the discovery of induced pluripotent stem (iPS) cells, they are so powerful that they can be tumorigenic (Takahashi and Yamanaka, 2006). On the other side we have multipotent cells, which are limited to the generation of tissues within the same germ layer but they are safer and indeed they have already been adopted to correct some of these malformations (Pittenger et al., 1999; Elliott et al., 2012).

Embryonic stem cells would be ideally positioned to build muscle tissues for children with congenital malformations (Thomson et al., 1998). However, besides their tumorigenic potential and the ethical issues, immunosuppressive treatment should also be adopted to avoid their rejection by the transplanted patient. It is believed that embryonic stem cells are less immunogenic, but this is only true if you consider them prior to differentiation. Once they are terminally differentiated and express all the major histocompatibility complexes, they would be rejected if immunosuppression therapies were not adopted. ES cells have been demonstrated to differentiate reliably to cardiomyocytes (Burridge et al., 2012), which have been successfully engineered to obtained cardiac microtissues (Thavandiran et al., 2013). However, the fully formed heart is composed of diverse cell lineages including myocytes, endothelial cells, vascular smooth muscle cells (SMC), and fibroblasts that derive from distinct subsets of mesoderm during embryonic development. As a consequence engineering of functional cardiac muscle for clinical application is still a major challenge. In this regard, ES cells can also be differentiated into distinct populations of SMC subtypes under chemically defined conditions (Cheung et al., 2012). As such, their ability to derive an unlimited supply of human cell types, including SMCs, could further accelerate applications of stem cells to regenerative medicine as well as disease modeling (e.g., patient-specific stem cells for exploring mechanisms of disease) (Cheung et al., 2014). PAX7-positive skeletal muscle progenitors can also be obtained from human and mouse ES cells opening the possibility of engineering autologous skeletal muscle in large quantity through the direct reprogramming of cells from children born with a malformation (Shelton et al., 2014).

On the opposite side of the picture there are the adult stem cells (Pittenger et al., 1999). Somatic stem cells can be expanded from different postnatal tissues and could be useful for therapy particularly in neonates and children where they are generally more abundant and probably more potent than in adults (Fulle et al., 2012). Classically the bone marrow contains, besides haematopoietic stem cells, mesenchymal stem cells, but somatic cells with different potentials can also be isolated and grown in good quantities. These cells can be used in an autologous setting avoiding immunogenic problems. As far as we know they are not tumorigenic, and their use does not raise any ethical issues (Bianco et al., 2008).

## Proof of concept for clinical applicability of RM

As an example of the potential of TE and RM technologies, 15 years ago a cover of Nature Biotechnology celebrated the first artificial bladder taking shape in dogs (Oberpenning et al., 1999). In those studies, the whole dome of the bladder was successfully replaced using smooth muscle and urothelial cells expanded from the recipient and this established the basis for treating the first patients affected by bladder exstrophy. The group, coordinated by Dr. Atala, described in 2006 in The Lancet a pilot study of seven patients who had received implanted tissue engineered bladders from 1998 onwards (Atala et al., 2006). Similar to the animal model, they reported the use of either collagen scaffolds seeded with cells or a combined polyglycolic acid (PGA)-collagen scaffold seeded with cells for bladder replacement. These engineered tissues were implanted with or without omental coverage. Patients reconstructed with engineered bladder tissue created with cell-seeded PGA-collagen scaffolds and omental coverage showed increased compliance, decreased end-filling pressures, increased capacities and longer dry periods over time (Atala et al., 2006). More recently, the same group showed that in 5 boys who had urethral defects, tubularised urethras could be engineered and remain functional in a clinical setting for up to 6 years (Raya-Rivera et al., 2011). A tissue biopsy was taken from each patient, and the muscle and epithelial cells were expanded and seeded onto tubularised polyglycolic acid:poly(lactide-coglycolide acid) scaffolds. Patients (range 10–14 years old), who had surgery between March 2004, and July 2007 were followed up until July 2010 showing maintenance of normal function and tissue architecture after biopsy (Raya-Rivera et al., 2011). However, as recently noted by Andersson (2014), despite encouraging proof of concept results, the more widespread applications of the bladder repair technologies awaits further preclinical investigation. Another example of the utility of somatic cells for TE and RM applications derives from the use of adult cardiomyocytes. In contrast to what was initially thought, cardiomyocytes can also be expanded from adult tissue and they have been used in in patients with ischaemic cardiomyopathy (Bolli et al., 2011). However, their numbers are limited and expansion may not be efficient enough to generate sufficient cell populations for engineering functional tissue. In addition, satellite cells, the skeletal muscle precursors, can be easily isolated and expanded. In fact, satellite cells have been used for cellular therapy and tissue engineering purposes in both synthetic and decellularised polymers in small and large animal models. Freshly isolated SCs showed a higher regenerative potential, with implemented proliferation and migration. They retain a high myogenic potential *in vitro* and more interestingly *in vivo* during the first few passages but they are unable to be expanded for longer in culture (Rossi et al., 2010). Within the muscle there are at least two other cell types, muscle associated but not somite-derived, that present a high myogenic potential. The mesoangioblasts, vessel-associated stem cells, express early endothelial markers, such as Flk-1, CD34, stem cell antigen 1 and VE (vascular-endothelial)-cadherin, but not late markers, like Von Willebrand factor (Cossu and Bianco, 2003). They can be expanded for several passages, are not tumorigenic and, even if they do not express the transcription factors Myf5 and MyoD, they can be easily induced toward myogenesis upon co-culture with myoblasts. Similarly, pericytes have also shown myogenic potential. They are, as the mesoangioblasts, vessel-associated progenitors, they do not express endothelial markers but they do express NG2 proteoglycan and alkaline phosphatase (ALP). Unlike the canonical myogenic precursors (SCs), pericyte-derived cells express myogenic markers only in differentiated myotubes, which they form spontaneously with high efficiency (Mitchell et al., 2010).

Given these initial successes and the possibilities they portend, why don't we always use adult stem cells? First, because the numbers of cells are small and they decrease with age. Second, these cells are multipotent not pluripotent, so they cannot give rise to all lineages. Finally, they can be exposed to virus and toxins during their lifetime. (Pittenger et al., 1999). That means that we have cells in our body that continuously accumulate deletions and mutations (Bianco et al., 2001). Our immune system normally destroys them, however if they are replicated in large numbers in the laboratory and transplanted back in the recipient they may be able to fight against our immune system and generate a tumor.

In 2006 a seminal paper published by Shinya Yamanaka described how some of the limitations of both embryonic and adult stem cells might be overcome (Takahashi and Yamanaka, 2006). His group found, first in mice and subsequently in humans, that pluripotent stem cells could be generated from their adult counterpart using defined transcription factors (Takahashi et al., 2007). The findings were confirmed by independent groups and it is now possible to derive induced pluripotent stem (iPS) cells using different methodologies (Zhao and Daley, 2008). iPS cells, when compared to ES cells, eliminate the immunogenic problem, so you can use them in an autologous setting, and they also reduce the ethical concerns. However, iPS are still tumorogenic and their clinical use has still not been adopted.

Amniotic fluid stem (AFS) cells should also be considered. They are distinct both from adult and embryonic stem cells, can be used in an autologous setting, their use is not controversial and they are not tumorogenic (Pozzobon et al., 2010). Moreover, they are more naïve than adult stem cells and can be superior both in terms of proliferation and differentiation. Isolation of stem cells from amniotic fluid is easy to perform, there's a low risk for the mother and the fetus and it is a widely accepted method for prenatal diagnosis. So, AFS cells are ideal for pre-natal and neo natal applications (Moschidou et al., 2013a). AFS cells, are immunoselected by the stem cell factor receptor c-kit (CD117) and give rise to lineages representing the three germ layers both *in vitro* and *in vivo* (de Coppi et al., 2007a). The cells express markers of all three germ layers, and endogenously express the important transcription factor OCT4, which maintains the pluripotency of ESCs. AFS cells are easily reprogrammed not only by DNA-integrating systems (Wolfrum et al., 2010), but also without any genetic manipulation by means of the histone deacetylase inhibitor, valproic acid (VPA) (Moschidou et al., 2012, 2013b). Both human and rodent AFS cells display multi-lineage potential (Ditadi et al., 2009) and can exert a beneficial paracrine action in models of bladder (de Coppi et al., 2007b), heart (Bollini et al., 2011), kidney (Sedrakyan et al., 2012), and lung (Grisafi et al., 2013) disease.

AFS cells could also have a role for *In utero* stem cell therapy (IUSCT) (Surbek et al., 2008). IUSCT in humans have been successful only for the treatment of congenital severe combined immunodeficiency (SCID). (Tiblad and Westgren, 2008) Rejection of allogeneic cells *in utero* could be at least partially explained by the migration of the *in utero* injected cells into maternal circulation and mounting of a rejection response, which could diminish the engraftment. This is most likely due in mice to activated maternal T cells which can cross the placenta in mice and destroy engrafted allogeneic cells (Nijagal et al., 2011). In order to avoid this response, stem cells matched to the mother could be used. Alternatively, in monogenic disease, AFS cells derived from the fetus could be used for therapy after genetic modification since they would not trigger an immunogenic response from either the fetus or the mother.

Regarding the application of AFS cells for the treatment of acquired muscle conditions, we and others have tested various disease models. In a mouse model of Spinal Muscular Atrophy with a muscular dystrophy appearance of the skeletal muscle (*HSA-Cre, SmnF7/F7* mice) receiving intravenous injection of a small number of AFS cells were able to survive with drastic improvement of their muscle force (Piccoli et al., 2012). Histopathological evaluation of the treated animals revealed integration of AFS cells not only in the skeletal muscle fibers, but also in the stem cell compartment of the muscle. Indeed secondary transplants of satellite cells (SCs) derived from treated mice indicated that AFS cells integrate into the muscle stem cell compartment and have long-term muscle regeneration capacity **indistinguishable** from that of wild-type-derived SC (Piccoli et al., 2012).

AFS cells however, have not been used clinically yet. Therefore, attempts to generate tissues or organs in the laboratory for the correction of congenital malformations has only been tried thus far using adult somatic cells.

## Developing TE strategies for the fetus and newborn

As summarized above, there has been a lot of research directed toward identifying cell source(s) with potential applications for improving TE and RM technologies. Another critical component of TE/RM approaches is the biomaterial component. Although, as noted in a recent review (Wolf et al., 2014), biomaterials/scaffolds alone are being actively pursued both pre-clinically and clinically for restoration of volumetric muscle loss (VML; injuries of sufficient magnitude to result in permanent functional and cosmetic deficits) injuries in adults, the focus in this review will be on TE and RM solutions for the fetus/neonate. In addition, several other excellent recent reviews are available that address more general aspects of TE/RM for skeletal muscle repair (Rossi et al., 2010; Juhas and Bursac, 2013; Mertens et al., 2014).

With respect to the explicit purpose of this report, any contemplated TE implant for the fetus or neonate would need to grow with the patient, we direct the remainder of this report to consideration of biomaterials/scaffolds that are being designed for the presence of a cellular component. This emphasis seems especially applicable to the large volume muscle tissue replacement required for correction of congenital anomalies in fetuses and newborns, as discussed herein. In this scenario, the biomaterial serves as a cellular delivery vehicle that can provide structure and appropriate environmental context and/or instructional cues for improved regeneration. Recent research in this area has begun to address the enormous possibilities this approach portends.

## Biomaterials for skeletal muscle TE and RM for the fetus and newborn

As noted in the discussion thus far, the vast majority of preclinical studies conducted to date for skeletal muscle tissue repair—that might eventually be applicable to fetal/neonatal tissue repair and replacement—have used adult somatic cells, and in particular, the focus has been on myogenic progenitor cells (i.e., satellite cells, myoblasts, myotubes). This is true for studies conducted both *in vitro* and *in vivo*. In that regard, much progress has been made in engineering skeletal muscle since the seminal work of Vandenburgh and colleagues on avian myocytes in 1988 (Vandenburgh et al., 1988). The pertinent literature in this area is still actively growing. Below we provide a thorough, though not exhaustive, review of the recent PubMed database related to cell-based skeletal muscle tissue engineering approaches. The goal was to review the literature and identify the major sources of tested biomaterials/scaffolds for TE-based skeletal muscle repair/replacement.

**Figure 1:**
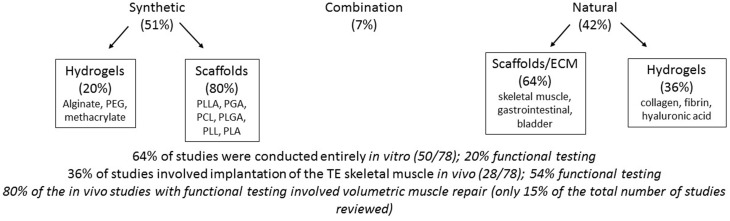
**Schematic summary of the main findings of literature review concerning tissue engineering approaches for muscle repair that combine cells plus a scaffold**.

As noted in Table 1 and schematically depicted in Figure 1, synthetic and naturally-derived biomaterials have been used with similar frequency for TE purposes. Of the studies reviewed, a naturally-derived biomaterial was used in roughly half of all studies conducted. Only a minority of studies have combined natural and synthetic biomaterials as part of the preferred scaffold configuration. Also of note, roughly 1/3 of the studies reviewed (25/78) have been conducted using C2C12 cells, which while more convenient to work with for a variety of reasons, lack clinical applicability. Thus, the discussion below emphasizes the use of primary cultures. So, how have these biomaterials been combined with myogenic cells to yield TE skeletal muscle?

**Table 1:**
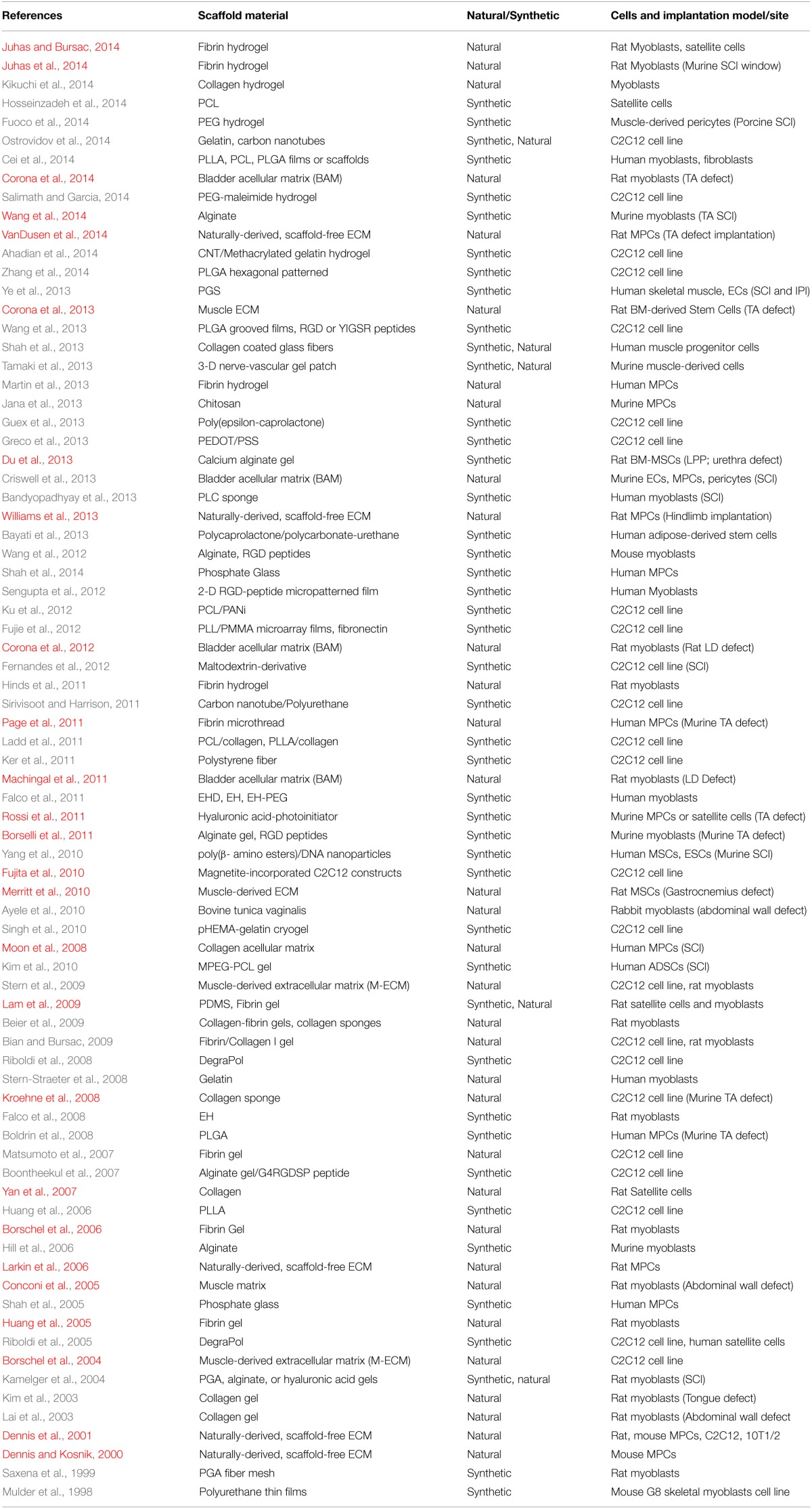
**Summary of literature on cell and biomaterial combinations used for tissue engineered skeletal muscle**.

*Red lettering indicates functional studies were performed on TE constructs implanted in vivo (i.e., contraction of engineered/retrieved constructs were evaluated. Highlighted in yellow are the engineered skeletal muscle tissues that were implanted, as well as the site and nature of implantation. Abbreviations: EH network: polymeric scaffolds (EH network) made from the cyclic acetal monomer, 5-ethyl-5-(hydroxymethyl)-β, β-dimethyl- 1,3-dioxane-2-ethanol diacrylate (EHD); PEG, polyethylene glycol; Poly-hydroxyethyl methacrylate (pHEMA)-gelatin cryogel scaffold; MPEG–PCL, methoxy poly(ethylene glycol) poly(3-caprolactone); PDMS, polydimethylsiloxane; PGA, poly glycolic acid; PLGA, poly-lactic-glycolic acid; PLC, L-lactide/epsilon-caprolactone copolymer; PLA, poly lactic acid; PLLA, poly (L-lactic acid); PLL, poly (L-Lysine); PMM, Poly (methyl methacrylate); PANi, polyaniline; PEDOT:PSS, poly(3,4-ethylenedioxythiophene):poly(styrene sulfonate); TA, tibialis anterior muscle; LD, latissimus dorsi muscle; SCI, subcutaneous implantation; MPCs, muscle progenitor/precursor cells; ADSCs, adipose-derived stem cells; MSCs, mesenchymal stem cells; ECs, endothelial cells*.

From an experimental perspective, three general approaches to *in vitro* TE skeletal muscle have been utilized thus far: (1) Cells embedded in a hydrogel, (2) Cells placed on or within a more structured/patterned scaffold, and (3) Cells placed in culture and allowed to develop their own extracellular matrix *in vitro* (scaffold-free, but resulting in a naturally-derived extracellular matrix). These cell-seeded scaffolds have been subjected to a variety of strategies including different culture media, incorporation of mechanical forces and electrical cues, as well as incubation times of distinct durations. The end result has been to produce myotubes and myofibers of varying lengths and diameters *in vitro*. A host of histological, immunochemical and molecular evaluations have been conducted to assess the phenotype of the TE skeletal muscle produced. However, only ≈30% of all studies conducted (either *in vitro* or *in vivo*) actually evaluated the contractile function of the resultant constructs (see Table 1 and Figure 1). Because the functional status of TE skeletal muscle, at all stages of the TE process, is critical to evaluating their potential clinical applications and experimental utility (e.g., as a screening tool for drugs *in vitro*), we will focus going forward on a discussion of those studies that measured function, especially those that did so *in vivo* or following implantation *in vivo*.

## Characteristics of TE skeletal muscle *in vitro*

One school of thought for development of TE skeletal muscle for *in vivo* implantation is to create constructs that are as similar to native skeletal muscle as feasible prior to implantation. An intrinsic benefit of this approach is that these same technologies may be applicable to drug screening for muscle toxins, as well as for treatment of muscle diseases and disorders. Thus far, however, all attempts to create TE skeletal muscle *in vitro* still result in a relatively immature/neonatal muscle phenotype, with respect to fiber diameter (generally <20 μM) and functionality (the degree of measured force following stimulation), as well as expression of embryonic myosin isoforms. In fact, absolute forces for TE skeletal muscle *in vitro* have typically ranged from as little as ≈1–30 μN (Borschel et al., 2004; Fujita et al., 2010), to 400–800 μN (Dennis and Kosnik, 2000; Dennis et al., 2001; Huang et al., 2005; Borschel et al., 2006; Lam et al., 2009). The specific force, when it has been measured (Dennis et al., 2001), has only been a fraction (<10%) of what might be considered normal for a mammalian/rodent skeletal muscle (250 kN/M^2^). The most complete functional analysis of force generation *per se* on TE skeletal muscle is that of Dennis and colleagues (Dennis et al., 2001). More recently, Larkin and colleagues (Williams et al., 2013; Mertens et al., 2014; VanDusen et al., 2014), as well as Bursac and colleagues (Perniconi et al., 2011; Juhas and Bursac, 2013, 2014; Juhas et al., 2014) have made significant improvements in both the phenotype and function (contractility) of TE skeletal muscle *in vitro*. Bursac, in particular, has shown that key aspects of excitation-contraction coupling (calcium transients) are intact, and moreover, that the constructs maintain the ability for myogenesis and regeneration *in vitro*. Both Bursac's biomimetic scaffolds (Juhas et al., 2014), as well as the SMUs (skeletal muscle units) of Larkin and colleagues (VanDusen et al., 2014) showed significant improvements in phenotype and contractility, as well as vascularization, following implantation *in vivo* for 1–4 weeks. These latter observations clearly point to the importance of the *in vivo* environment for enhanced maturation and function of TE skeletal muscle, even when TE muscle begins to more closely approximate native muscle with respect to excitation-contraction coupling and force generation. However, when thinking about this approach more broadly, it seems plausible that these more immature phenotypes may not be as large a barrier to TE skeletal muscle repair and replacement in the fetus and newborn, as they may be for skeletal muscle repair and replacement of VML injuries in adult mammals.

## Implantation of TE skeletal muscle

As detailed in Table 1, fewer than 36% (28/78) of all the studies we reviewed involved implantation of TE skeletal muscle constructs *in vivo*. These implantations were either subcutaneous or in a model of VML injury. The remaining 16 (57%) *in vivo* implantations were placed in a model of VML injury to assess restoration of muscle tissue volume and/or function. These are each briefly described below.

## *In situ* implantation of TE skeletal muscle

Of the 28 studies that included *in vivo* implantation, 12 (43%) were implanted subcutaneously or otherwise *in situ* (e.g., rat hindlimb), essentially using the body as a “bioreactor” to evaluate the impact of the *in vivo* environment on TE muscle maturation. However, only 3/12 studies actually evaluated contractile function (Moon et al., 2008; Williams et al., 2013; Juhas et al., 2014). Importantly, as alluded to above, in all of those (3) studies *in vivo* implantation was found to enhance muscle maturation and function.

## Skeletal muscle TE for improved regeneration of VML defects *in vivo*

The explicit goal of TE skeletal muscle for the fetus/neonate is to develop strategies that can repair or regenerate congenital anomalies. Importantly, the magnitude of muscle regeneration required in the VML rodent models is a reasonable approximation of the requirement of any TE/RM strategy in the fetus/neonate that would also be sufficient to accommodate growth of the fetus/neonate. Thus, another approach to TE skeletal muscle for repair and replacement *in vivo*, is to develop constructs that mainly mature *in vivo*. In contrast to *in vitro* TE approaches, many of these constructs lack the functional characteristics of even immature skeletal skeletal muscle (i.e., contraction; see above for details), but contain various combinations of satellite cells, myoblasts, myotubes, etc., on a cell delivery vehicle that will subsequently leverage the existing *in vivo* environment to provide the required key components for accelerated and/or enhanced functional regeneration in the scenario of VML injury.

In this regard, 16 studies evaluated implantation of a TE muscle construct in a VML defect *in vivo* (most commonly, surgically created defects), where by definition, there was no improvement expected in the absence of repair. In 75% of those studies functional outcomes were evaluated. Interestingly, with respect to surgically-created VML injuries to the legs (eight different studies), despite the distinct approaches that have been tried thus far (implantation of fibers in a hydrogel (Rossi et al., 2011), implantation of myoblasts on a fibrin microthread (Page et al., 2011), scaffold implantation with subsequent stem cell injection (Merritt et al., 2010; Corona et al., 2013), implantation of SMUs (VanDusen et al., 2014), bioreactor preconditioned myoblasts and myotubes (Machingal et al., 2011; Corona et al., 2012, 2014) in all cases, there were residual functional deficits, generally in the 20–30% range. Such an observation, albeit on a very small sample size with significant differences in models, muscles and measures, points out both the incredible promise of TE/RM for VML injury, as well as the limitations of current technologies and the need for standardized animal models and physiological measures.

## Potential clinical applications to neonates

We are not aware of any current or proposed clinical trial for the use of an RM/TE technology in the treatment of a craniofacial VML injury in the fetus or newborn. However, our group has been pursuing a tissue engineered muscle repair (TEMR) technology for clinical applications to craniofacial reconstruction and repair. We have been using the rodent latissimus dorsi (LD) muscle as a model system. The LD muscle has long-standing clinical utility (surgical reconstruction, heart wrap, etc.,) and further, is a relatively thin, sheet-like muscle that is morphologically analogous to the muscles in the face (i.e., muscles of mastication). The TEMR constructs have been implanted in a surgically created VML injury (i.e., excision of 50% of the LD muscle). These constructs are created by seeding myoblasts on a bladder acellular matrix (BAM), and subjecting the construct to cyclic mechanical preconditioning (10% stretch) in a bioreactor prior to implantation of a construct containing myoblasts and myotubes in a unidirectionally organized monolayer into the LD VML injury. As alluded to above, TEMR implantation is associated with restoration of significant functional capacity (60–70% recovery of contractile force) in athymic nude mice within 2 months of implantation (Machingal et al., 2011; Corona et al., 2012). This recovery appears to involve, at least to some extent, regeneration of a portion of the muscle fibers that were surgically removed.

Thus, building on the preclinical development of the TEMR technology, Christ and colleagues at UT-Houston (Drs. Mark Wong and Phil Freeman) have identified a craniofacial muscle-only defect (secondary revision of cleft lip), which represents a VML injury that might be effectively treated by this first generation TEMR technology. The study that will be proposed would be to address secondary revision of unilateral cleft lip (UCL) in adults. If successful though, these studies could have important implications for neonates as well. In fact, clefts of lip and the palate are among the most common congenital defects observed, with a frequency of about 1.7 per 1000 liveborn babies (Mossey et al., 2009), in which the orbicularis oris muscle has been shown to be deficient in both volume and function. In addition, secondary repair of UCL is necessary in a significant percentage of patients for correction of both functional and cosmetic deformities. It is conceivable, that TE approaches, such as the TEMR technology, may find utility for this clinical application, as implantation will occur in a fresh surgical wound bed in healthy subjects and is readily scalable to construct tested in rodents. Discussions are currently ongoing with the US Federal Drug Administration (FDA) to this end.

## Summary

Clearly there is certainly much to be excited about with respect to the potential applications of TE skeletal muscle for clinical applications to the fetus/newborn. Nonetheless, there is much work still to be done. In short, overall, too few functional assessments are being performed, as is too little work in relevant animal models. In addition, there are numerous biomaterials, animal models, muscles, time points, cell types, etc., that have been utilized thus far, and therefore, there is a need for standardization of animal models and functional measures to permit more direct comparisons of different approaches in similar VML injuries.

## Conclusion

In conclusion regenerative medicine and tissue engineering are changing the way we think about how we might 1 day treat patients born with serious congenital malformations involving muscle tissue. However, the science still needs time to better understand mechanism of action(s) responsible for improved functional regeneration, as well as the best cell(s) and biomaterial(s) and/or their combinations for maximizing the rate and magnitude of functional regeneration. In addition, we still must determine the safety profile of stem cell products and biomaterials prior to clinical applications. Nonetheless, the advances are coming rapidly along, and we need to be informed, educated and prepared to utilize these techniques for the benefit of patients, as soon as they are available.

### Conflict of interest statement

The authors declare that the research was conducted in the absence of any commercial or financial relationships that could be construed as a potential conflict of interest.
